# Transient Changes in Serum CEA, CA19-9, CRP, YKL-40, and IL-6 during Adjuvant Chemotherapy and Survival of Patients with Colorectal Cancer

**DOI:** 10.3390/ijms24076753

**Published:** 2023-04-04

**Authors:** Kaisa Lehtomäki, Eetu Heervä, Pirkko-Liisa Kellokumpu-Lehtinen, Harri Mustonen, Tapio Salminen, Heikki Joensuu, Kethe Hermunen, Mogens Karsbøl Boisen, Julia Sidenius Johansen, Caj Haglund, Pia Osterlund

**Affiliations:** 1 Faculty of Medicine and Health Technology, Tampere University, Arvo Ylpön katu 34, 33520 Tampere, Finland; 2 Department of Oncology: Tays Cancer Center, Tampere University Hospital, Teiskontie 35, 33520 Tampere, Finland; 3 Department of Oncology, Turku University Hospital, Hämeentie 11, 20520 Turku, Finland; 4 Department of Oncology, University of Turku, Kiinanmyllynkatu 10, 20520 Turku, Finland; 5 Research, Development and Innovation Center, Tampere University Hospital, Teiskontie 35, 33520 Tampere, Finland; 6 Department of Surgery, Helsinki University Hospital and University of Helsinki, Haartmaninkatu 4, 00290 Helsinki, Finland; 7 Research Programs Unit, Translational Cancer Medicine Program, University of Helsinki, Haartmaninkatu 8, 00290 Helsinki, Finland; 8 Department of Oncology, Helsinki University Hospital and University of Helsinki, Haartmaninkatu 4, 00290 Helsinki, Finland; 9 Department of Oncology, Herlev and Gentofte Hospital, Copenhagen University Hospital, Borgmester Ib Juuls vej 1, DK-2730 Herlev, Denmark; 10 Department of Medicine, Herlev and Gentofte Hospital, Copenhagen University Hospital and University of Copenhagen, Borgmester Ib Juuls vej 1, DK-2730 Herlev, Denmark; 11 Institute of Clinical Medicine, Faculty of Health and Medical Sciences, University of Copenhagen, Blegdamsvej 3, DK-2200 Copenhagen, Denmark; 12 Department of Gastrointestinal Oncology, Tema Cancer, Karolinska Universitetssjukhuset, Eugeniavägen 3, 17176 Solna, Sweden; 13 Department of Oncology-Pathology, Karolinska Institutet, Solnavägen 1, 17177 Solna, Sweden

**Keywords:** colorectal cancer, CEA, CA19-9, IL-6, C-reactive protein, YKL-40, biomarker, adjuvant chemotherapy, transient increase

## Abstract

Serum carcinoembryonic antigen (CEA) is frequently monitored to detect colorectal cancer (CRC) recurrence after surgery. The clinical significance of transiently increased CEA during adjuvant chemotherapy is poorly understood. Serum CEA, CA19-9, CRP, YKL-40, and IL-6 were measured before, during, and after adjuvant 5-fluorouracil-based chemotherapy in the randomised LIPSYT study population. The biomarker kinetic patterns were classified into three groups: no increase, a transient increase (≥10% increase followed by a decrease), and a persistent increase during the adjuvant treatment, and the associations of these patterns with disease free-survival (DFS) and overall survival (OS) were investigated by using Cox regression analyses. The findings were validated in two single-centre cohorts that received modern adjuvant chemotherapy. A transient increase in CEA occurred in about a half of the patients during chemotherapy, in all the cohorts. The patients with a transient increase had a roughly similar DFS and OS to the patients with no increase, and a more favourable survival compared to the patients with a persistent increase. In the LIPSYT cohort, the hazard ratio was 0.21 for DFS (CI_95%_ 0.07–0.66) and 0.24 for OS (CI_95%_ 0.08–0.76). Transient increases in CA19-9 and YKL-40 tended to be associated with a favourable survival. A transient increase in CEA during adjuvant chemotherapy is associated with a favourable survival when compared with a persistent increase.

## 1. Introduction

Patients who have undergone surgery for localised colorectal cancer (CRC) and those who have a high risk for recurrence are generally considered for adjuvant chemotherapy [1]. After this adjuvant treatment, the patients are often followed up by using computed tomography (CT) and serial measurements of their serum carcinoembryonic antigen (CEA) to detect recurrence early. In international guidelines, serum CEA is the only tumour biomarker that is recommended for monitoring purposes during and after adjuvant therapy [2,3]. Preoperatively or postoperatively elevated CEA predicts an impaired disease-free survival (DFS) and overall survival (OS) [4,5]. The serum half-life of CEA is about 25 days, and concentrations increasing above the baseline value suggest a persistent or progressive disease [2,3]. When a threshold of 5 µg/L for the serum CEA was used, the pooled sensitivity of the CEA for CRC recurrence was 71%, with a specificity of 88%; however, approximately 25% of recurrences occur without an accompanying increase in the serum CEA concentration [6]. Therefore, surveillance might be augmented by using other prognostic and/or predictive biomarkers that, either alone or with CEA, could recognize the patients that are at a high risk for recurrence [7,8].

Carbohydrate antigen 19-9 (CA19-9) is another widely used biomarker that may be elevated at the time of CRC recurrence, but this occurs in only 21% to 36% of patients [9,10]. Thus, the prognostic value of serum CA19-9 alone is insufficient [7,9,11] and, therefore, it is usually measured together with CEA [12,13]. Elevated acute-phase biomarkers, such as the C-reactive protein (CRP) and YKL-40 (also called chitinase-3-like-1 protein, CHI3L1) [14], are associated with a poor prognosis for various types of cancer, including CRC [15,16,17,18]. YKL-40 is a glycoprotein with a role in inflammation, the remodelling of the extracellular matrix, angiogenesis, metastasis, and protection against apoptosis [14,15]. Interleukin 6 (IL-6), a cytokine that is produced during acute and chronic inflammation, triggers several signalling cascades in cancer that affect cell proliferation and survival, the inhibition of apoptosis, and the induction of anti-cancer drug resistance. A high serum IL-6 concentration is associated with a poor survival in metastatic CRC (mCRC) [18,19,20].

Despite intensive research, there are few validated biomarkers available that predict the benefit that is derived from adjuvant chemotherapy in stage II or III CRC [21]. For example, circulating tumour DNA (ctDNA)-based technologies have raised enthusiasm, but some challenges in their methodologies have to be overcome before their incorporation into routine follow-up within the adjuvant setting [22,23]. Therefore, further research on older biomarkers such as CEA is necessary. CEA change pattern rather than single measurements, seem important when evaluating CEA during and after chemotherapy [4,7,8]. Most evidence about CEA’s change patterns, during and after chemotherapy, comes from studies that have been carried out in mCRC, where the treatment response can be evaluated with imaging [24,25,26,27,28], which is not feasible in the adjuvant setting. Only a few studies have investigated CEA kinetics during adjuvant chemotherapy, and these have been relatively small in size, with less than 100 individuals in each study [29,30,31]. Interestingly, a transient increase in CEA may occur in some patients [29,31]. A study with 61 patients concluded that the patients who had a transient elevation in their CEA during adjuvant chemotherapy had similar OS compared with the patients who had no CEA increase, and both groups had a more favourable survival than the patients whose CEA increased persistently (36). However, the prognostic significance of this transient increase in serum CEA during adjuvant chemotherapy is not well understood.

The primary aim of the present study was to investigate the patterns of change in serum CEA, CA19-9, CRP, YKL-40, and IL-6 during adjuvant chemotherapy, in three patient cohorts: the LIPSYT study cohort from Helsinki University Hospital, a validation cohort from Tampere University Hospital (TAUH), and a second validation cohort from Turku University Hospital (TUH). The patients in these three cohorts had undergone surgery for CRC with a curative intention. We also aimed to find out whether the serum concentrations of these proteins are potential prognostic biomarkers for survival.

## 2. Results

### 2.1. Patient Characteristics

The LIPSYT study cohort included 131 patients that were randomised to a bolus vs continuous infusion 5-fluorouracil-based adjuvant chemotherapy for radically resected stage II-IV CRC. The retrospective Tampere University Hospital (TAUH) and Turku University Hospital (TUH) validation cohorts included 346 patients and 279 patients, respectively, who received oxaliplatin-based or 5-fluoruracil-based adjuvant chemotherapy for radically resected stage II-IV CRC. A flow diagram for the CEA in the three cohorts is shown in Figure S1A. The patient and tumour characteristics were generally similar in the LIPSYT study cohort and two validation cohorts, except for the median age of the trial patients being younger (60 years) compared to the TAUH and TUH validation cohorts (67 years in both cohorts, Table 1). In all the cohorts, the patients with stage IV metastatic disease had a radical metastasectomy prior to starting chemotherapy (mostly liver resections). The patients with a rectal primary tumour received chemoradiotherapy more often in the LIPSYT cohort compared to the patients in the two validation cohorts. The covariables that were included in the multivariate analysis are shown in Table 1.

### 2.2. CEA Change Groups

The CEA was measured before, once mid-adjuvant, and approximately two months after the adjuvant treatment. The biomarker kinetic patterns were classified into three groups: no increase, a transient increase (a ≥10% increase followed by a decrease), and a persistent increase. A transient increase in the serum CEA concentration occurred in 42 (46%), 176 (51)%, and 197 (71%) out of the 91, 346, and 279 patients in the LIPSYT (Figure S2), TAUH, and TUH cohorts, respectively. A persistent increase occurred in 17 (19%), 98 (28%), and 50 (18%) of the patients, and no increase occurred in 32 (35%), 72 (21%), and 32 (11%) (Table 2) of the patients. The patient demographics for the three kinetic groups are provided in Table S1.

### 2.3. CEA Change and Survival

The median reverse follow-up time for the patients in the LIPSYT study cohort was 11.9 years, and 4.2 years and 4.1 years for those in the TAUH and the TUH cohorts, respectively. The numbers of the CRC recurrences, deaths, and deaths that were considered to result from an intercurrent cause in each cohort are provided in Table 3.

**Figure 1:**
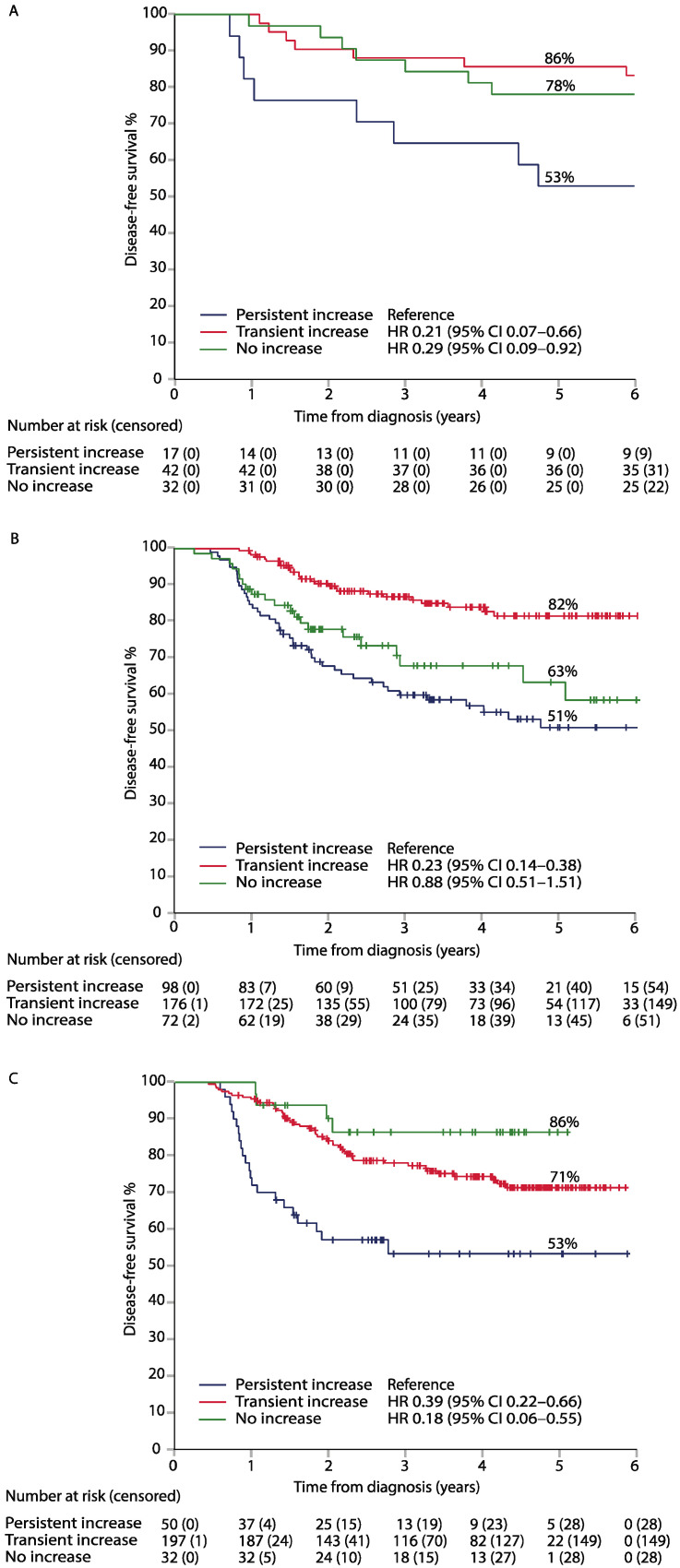
Disease-free survival (DFS) in the LIPSYT study (panel (**A**)), TAUH (panel (**B**)), and TUH (panel (**C**)) cohorts, according to CEA dynamics within reference during adjuvant therapy.

**Figure 2:**
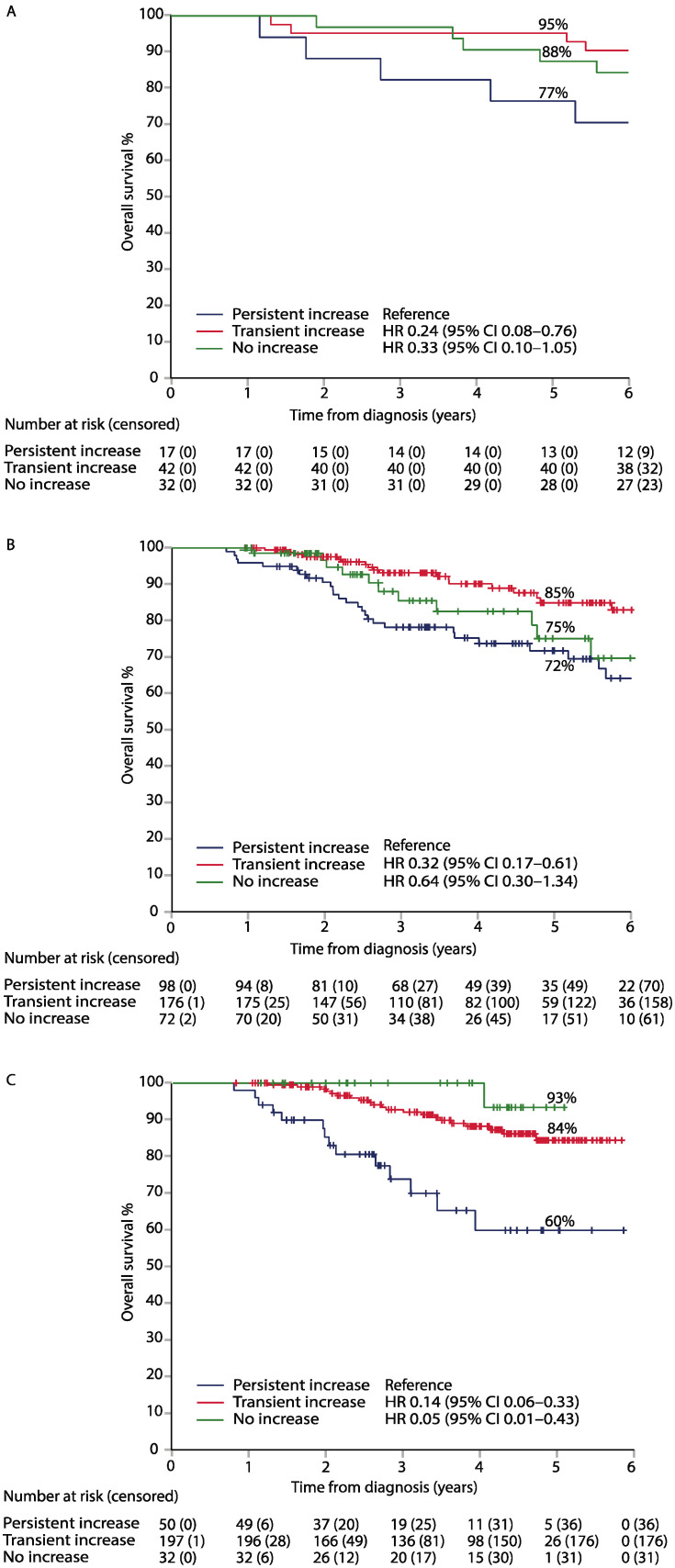
Overall survival (OS) (panel (**A**)) in the LIPSYT study, TAUH (panel (**B**)), and TUH (panel (**C**)) validation data, according to CEA dynamics within reference during adjuvant therapy.

The patients with a transient increase in their serum CEA had a longer DFS than those with a persistent increase in all three cohorts, with adjusted HRs of 0.21 (95% CI, 0.07–0.66), 0.23 (95% CI. 0.14–0.38), and 0.39 (95% CI, 0.22–0.66) in the LIPSYT, TAUH, and TUH cohorts, respectively (Figure 1, Table 4). The DFS in 86 stage II-III LIPSYT study patients (5 patients with stage IV disease were excluded) was similar to the adjusted HR for a transient increase of 0.17 (0.05–0.59). Similarly, the patients with a transient increase in their serum CEA had a longer OS than the patients with a persistent rise in their serum CEA, with adjusted HRs of 0.24 (95% CI, 0.08–0.76), 0.32 (95% CI, 0.17–0.61), and 0.14 (95% CI, 0.06–0.33) in the LIPSYT, TAUH, and TUH cohorts, respectively (Figure 2, Table 4). The patients with a persistent increase in their serum CEA concentration had the poorest DFS and OS in all three cohorts.

### 2.4. CA19-9, CRP, YKL-40, and IL-6

A transient increase in CA19-9 was observed in 6 (12%) out of 50 patients in the LIPSYT cohort and 48 (46%) out of 104 patients in the TAUH cohort, as was an increase in CRP in 21 (18%) out of 119 patients and 25 (9%) out of 283 patients, respectively. YKL-40 was transiently increased in the LIPSYT cohort in 52 (57%) out of the 92 patients with information available, as was IL-6 in 42 (43%) out of 97 patients (Figure S1B; Table 2).

The patients with a transient increase in their serum CA19-9, CRP, YKL-40, and IL-6 tended to have a favourable DFS and OS compared to the patients with a persistent increase in these serum biomarkers, with the point estimates of the HRs being smaller than 1 for all the survival analyses (Table 4, Figures S3–S6). In several of the analyses, the 95% confidence interval crossed 1, possibly reflecting the limited numbers of the patients and events in these analyses. When the survival of the patients with no increase in their serum CA19-9, CRP, YKL-40, and IL-6 was compared to the DFS or OS of the patients who had a persistent increase in these serum biomarkers, all except one of the point estimates of the HR were smaller than 1, but all the HR 95% confidence intervals crossed 1 (Table 4).

### 2.5. Association between Biomarker Kinetic Groups and Adverse Events during Adjuvant Therapy in the LIPSYT Study

Since a transient increase or persistent increase in a serum biomarker concentration might be associated with chemotherapy-related liver toxicity or other reasons [6], we investigated the associations between the biomarker change patterns and the blood cell counts, blood chemistry, and adverse events that were captured in the LIPSYT study. We did not find any significant associations between the three biomarker kinetic groups and the liver function tests (alkaline phosphatase, alanine aminotransferase, and aspartate aminotransferase), kidney function tests (creatinine, sodium, and potassium), or other laboratory tests that were performed, or with the adverse events that were captured during the trial (Table S2).

This section may be divided by subheadings. It should provide a concise and precise description of the experimental results, their interpretation, and the experimental conclusions that can be drawn.

## 3. Discussion

When arranging the CRC patients according to their patterns of serum biomarker concentrations during adjuvant chemotherapy, we could identify the groups with a reduced risk for recurrence and improved survival. The patients with a transient increase in their serum CEA during adjuvant therapy, with the rise being modest and mostly within the reference range, had a 61% to 79% lower risk for a DFS event and a 68% to 86% lower risk of death than the patients with a persistent increase in their CEA, when adjusting for the several prognostic factors that were observed in the three independent study cohorts. Similar trends were observed for the other biomarkers that were investigated.

The sensitivity of a single CEA test for detecting CRC recurrence is insufficient, and lowering the threshold of the test positivity from 5 µg/L to 2.5 µg/L leads to increased numbers of false positive results [8]. Therefore, it has been proposed that, rather than making treatment decisions based on a single CEA measurement, they should be made based on successive measurements and CEA kinetics over time [32]. Persistently increasing serum CEA levels indicate cancer progression and reflect an increase in the tumour burden, both in patients with localized CRC and those with metastatic CRC [4,26,27,33]. According to international guidelines, persistently increasing serum CEA levels in three subsequent measurements should trigger imaging studies and colonoscopy in CRC patients resected with a curative intent [2,3,34,35], in a manner analogous with serum PSA measurements in patients with prostate cancer [36]. However, little is known about the clinical significance of the serum biomarker changes that are relatively small and often occur within the reference range in patients with CRC. The current results suggest that a persistent increase in the serum CEA is a signal for a poorer prognosis compared to a transient increase.

The reasons for a transient CEA increase during adjuvant treatment and its association with a favourable prognosis remain speculative. An early transient elevation or surge of CEA and other biomarkers has been described during first-line chemotherapy for mCRC [24,25], which could represent an efficient killing of the cancer cells and the subsequent release of CEA into the blood stream. Several studies have reported that 10% to 15% of mCRC patients have a transient increase in their serum CEA at the initiation of oxaliplatin-based or irinotecan-based chemotherapy [24,28,37,38]. This increase occurs 2 to 4 weeks after the initiation of the chemotherapy and can last for up to 6 to 12 weeks [28,37,39]. A transient increase in the serum CEA appears to be associated with a favourable response to chemotherapy [24,28,37,38,39] and the survival [24] of patients with mCRC. Therefore, the monitoring of serum CEA is recommended after 12 weeks from the treatment initiation, and CEA may be considered to be an intermediate endpoint for clinical trials on mCRC [26]. In the adjuvant setting, a transient increase could represent a greater occult tumour burden and a higher chemotherapy tumour lytic effect [29,30,40]. Cancer differentiation is associated with serum CEA levels, as 80% of the patients with well-differentiated CRC, but only 60% of the patients with poorly-differentiated cancer, had elevated CEA [4,6], but the grade was also not associated with the serum CEA change pattern in our cohorts. The transient CEA increase that we observed could thus, in part, reflect the killing of the tumour cells, but the late timing of the mid-adjuvant blood sample (≥3 months after starting chemotherapy) suggests that other mechanisms may also contribute.

The transient increase could also be related to the effects of chemotherapy on the normal gastrointestinal or liver cells [29]. As CEA is primarily metabolized in the liver, hepatic dysfunction or biliary obstruction can be associated with CEA elevation [6]. There are also multiple other causes and conditions that have been associated with CEA elevation, such as the presence of non-colorectal cancer [4], hypothyroidism [41], inflammatory bowel conditions [42], COVID-19 infection [43], cardiopulmonary bypass [44], and haemodialysis [45]. In addition, up to 19% of smokers, with no evidence of malignancy, had elevated levels of serum CEA [6]. The patients who were eligible for the adjuvant therapy rarely had such comorbidities that could affect the present findings. We investigated inflammatory bowel diseases in our cohorts, but found no associations with the CEA groups. The smoking statuses, captured only in the TUH cohort, were not associated with the CEA group either. All the patients with elevated baseline CEA levels were excluded from the study, thus including some smokers. In the LIPSYT study, we looked at liver and kidney function tests, coagulation factors, and full blood counts in a controlled sampling setting and found that they had no associations with the CEA group. We also evaluated the adverse events of the gastrointestinal tract, the mucosa, and the skin, as well as the worst haematological or non-haematological toxicity that was recorded, and none of these correlated with a transient or persistent increase in the serum CEA. We cannot, however, exclude the possibility that CEA may be released from normal cells into the blood stream, resulting in a transient increase in the serum CEA during chemotherapy.

To the best of our knowledge, few prior data are available regarding the CEA change patterns in patients with CRC that have been treated with adjuvant therapy. Only one of the three small retrospective studies (34–36) has reported on the patient survival outcomes with adjuvant treatments in a group with a transient increase in their serum CEA. In accordance with the current findings, in Lawrence et al., the 5-year overall survival was higher in the transient CEA elevation group than in the persistent elevation in CEA group, 95% vs. 43%, respectively (36). To our knowledge, there is no generally accepted definition for a transient serum biomarker increase, which complicates the comparison of the current findings with prior observations. We used the definition of an increase of 10% from the pre-adjuvant value, in which the change was greater than the intra-assay or inter-assay variation that was observed with the methods used. With this definition, we observed a transient increase in the CEA in 46% to 71% of the patients, while Lawrence et al. found a transient increase in 33% of the patients, defining the increase as twice the within-patient standard deviation [31]. An arbitrary increase or decrease of >0.5 µg/L has also been used [29], and sometimes this definition may be lacking [30]. In mCRC studies, a transient CEA rise or fall of 15–20% from the prechemotherapy value has been used [24,28,37,38,39], but this definition was not applicable to our study.

We found similar trends between a transient increase in serum CA19-9, CRP, YKL-40, and IL-6 and a good prognosis, as with the serum CEA, raising the question of whether a transient increase in a serum biomarker level during chemotherapy could be associated with a favourable survival in other types of human cancer. To our knowledge, there are only a few studies that have investigated the transient increases in biomarker levels other than CEA during chemotherapy. A transient increase in the serum CA19-9 during a treatment for mCRC [38] and in serum TIMP-1 during an adjuvant treatment of CRC [30] have been described, but these findings were not correlated with the outcome. A transient increase in the serum CEA and CA15-3 may occur in patients with metastatic breast cancer that are receiving systemic chemotherapy [46], and similar surges of serum biomarkers have been demonstrated in patients with a hepatocellular carcinoma regarding α-fetoprotein [47], in patients with a testicular germ cell tumour with human chorionic gonadotropin [48], and in patients with metastatic gastric cancer with CEA and CA19-9 [49], mostly predicting the clinical benefits of chemotherapy.

This study has some limitations. Its main limitation is the small size of some subgroups, which was aggravated by the exclusion of patients with an abnormal pre-adjuvant biomarker concentration and those who had a cancer recurrence during the 8 months of adjuvant therapy, resulting in a limited statistical power of some of the analyses. The LIPSYT study recruited patients from 1997 to 2001, when oxaliplatin was not yet generally available, and radiotherapy, surgery, and cancer imaging were different from today. Yet, we confirmed many of its findings in two more recent TAUH and TUH validation cohorts from two university hospitals, in which the patients were treated according to the current standards and over half of the patients received oxaliplatin-based therapy. However, the follow-up time was significantly longer in the LIPSYT study, which was a possible source of bias. In addition, while we cannot rule out chance variation, the 10% cut-off that was chosen is compatible with the 1% to 5% intra-assay variation and the 3% to 9% inter-assay variation in the CEA assays of the accredited laboratories. The strengths of the study include its mature patient follow-up data, with no patients lost to follow-up. Additionally, clinical and radiologic examinations were performed every 1 to 2 years, and, therefore, asymptomatic cancer recurrences in patients with normal CEA and CA19-9 were also detected. The biomarkers that were studied are generally available and the blood sampling was standardised in all the cohorts. Mid-adjuvant samples were collected from all the patients, thus reducing the risk of a sampling bias.

## 4. Materials and Methods

### 4.1. Patients

We evaluated the biomarkers within the context of the LIPSYT study, which was an open-label, prospective, two-by-two factorial design, randomised phase III, single-institution study on patients with radically resected (R0 or R1 resection) stage II, III, or IV CRC (ISRCTN98405441). The accrued patients received adjuvant chemotherapy at the Department of Oncology, Helsinki University Hospital, Finland, from 1997 to 2001. The primary aim of the trial was to assess the treatment tolerability, and the secondary aim was to study cancer biomarkers. The patients were randomly allocated to receive 6 months of adjuvant chemotherapy, consisting of 5-fluorouracil (5-FU) and leucovorin (LV) that were administered either as bolus injections (the Mayo regimen) or as continuous 5-FU infusions (simplified de Gramont regimen) [50]. The study was conducted according to the Declaration of Helsinki and the study protocol was approved by an institutional review board at the Helsinki University Hospital. All of the study’s participants provided signed informed consent prior to the initiation of the study’s related procedures.

These serum biomarkers were studied also in two independent, retrospective validation cohorts consisting of CRC patients who were treated with adjuvant chemotherapy. One of the cohorts consisted of patients that were treated at the Department of Oncology, at Tampere University Hospital (TAUH), Tampere, Finland, from 2012 to 2020, and the other cohort consisted of patients that were treated at the Turku University Hospital (TUH), Turku, Finland, from 2011 to 2018, as described elsewhere [51]. The patients had radically resected (R0 or R1 resection) stage II, III, or IV CRC. The patients with stage IV cancer underwent a metastasectomy with a radical (R0) liver or lung resection and received adjuvant chemotherapy after this resection; none had received preoperative therapy. The adjuvant chemotherapy regimens were fluoropyrimidine-based, mostly consisting of capecitabine, with or without oxaliplatin. Hospital permission for the collection of the data from these retrospective cohorts was obtained (an ethics committee approval is not required for retrospective studies in Finland).

In all three cohorts, the planned duration of the adjuvant chemotherapy was 6 months. Patients with at least 3 months of this adjuvant chemotherapy were eligible for the current study. All the patients had a CEA measurement performed mid-adjuvant therapy. We excluded the patients who had a recurrence during the adjuvant therapy, or within 2 months after the completion of the adjuvant therapy (i.e., before collecting the post-adjuvant blood sample for the biomarker analysis). The CONSORT diagrams showing the reasons for these patient exclusions from the current analysis are provided in the Supplementary Materials (Figure S1A,B).

### 4.2. Blood Sampling

Serum CEA, CA19-9, CRP, YKL-40, and IL-6 were measured longitudinally within the LIPSYT study patient population. The serum CEA was also monitored in both the validation cohorts (the TAUH cohort and TUH cohort), whereas the serum CRP and CA19-9 were available only for the TAUH cohort. The CEA, CA19-9, and CRP were measured as part of the standard clinical routine in all three cohorts, whereas the IL-6 and YKL-40 were measured post hoc (only in the LIPSYT cohort).

In total, three blood samples were collected for the biomarker assays. The baseline blood sample was collected postoperatively, before initiating the adjuvant chemotherapy (“the pre-adjuvant sample”). Patients with a baseline biomarker value above the upper limit of normal were excluded from the statistical analyses of the markers (Figure S1). The second blood sample (“the mid-adjuvant sample”) was collected after approximately 3 months of chemotherapy, and the third sample (“the post-adjuvant sample”) was collected approximately 2 months after the date of completion of the adjuvant chemotherapy (approximately 10 months from the date of surgery). The serum CEA and CA19-9 were not routinely measured in the middle of the adjuvant therapy during the early patient accrual period, in any of the cohorts.

In the LIPSYT study, blood was drawn in the morning after overnight fasting, whereas in the validation cohorts, overnight fasting before the blood sampling was not required. In all three patient cohorts, the blood samples that were collected during the adjuvant therapy were taken immediately prior to the initiation of the next chemotherapy cycle [52].

### 4.3. Serum Biomarker Assays

The serum CEA was analysed using an immunoenzymatic assay, either with a Siemens Atellica IM assay (Erlangen, Germany) or a Bayer Immuno assay (Erlangen, Germany), from the samples that were collected in the LIPSYT study. The lower level of quantitation was <0.5 µg/L (Siemens) or 1 µg/L (Bayer), the intra-assay coefficient of variation (CV) was 5% in each assay, and the inter-assay CV was 9% (Siemens) or 7% (Bayer). The serum CA19-9 was measured using an immunoenzymatic assay (Bayer Immuno, Tarrytown, NY, USA), and the serum CRP using an immunoturbidimetric method (Roche Diagnostics, Mannheim, Germany) at the HUSLAB laboratories, Helsinki University Hospital. The lower level of quantitation was 0.8 kU/L for the CA19-9 and 5 mg/L for the CRP, the intra-assay CVs were <2% and 4.1%, and the inter-assay CVs were <3.0% and 3.2%, respectively.

Serum YKL-40 and IL-6 concentrations were determined from sera that was stored at −20 °C until the analysis. The analyses were performed in duplicate, using an enzyme-linked immuno-sorbent assay (ELISA). The YKL-40 was assessed using a MicroVue YKL-40 ELISA (Catalogue #8020, Quidel, Santa Clara, California, CA, USA), and the IL-6 with an IL-6 ELISA (Catalogue no. HS600, R&D Systems, Abingdon, UK), according to the manufacturer’s instructions. The lower level of quantitation was 20 ng/mL for the YKL-40 and 0.01 pg/mL for the IL-6, the intra-assay CVs were <5% and <8%, and the inter-assay CVs were <6% and <11%, respectively [53,54].

In the validation cohorts, the serum or plasma CEA was measured using an immunoturbidimetric method, either with a Roche Cobas (Pleasanton, California, USA; the TAUH cohort) or Roche Diagnostics (Mannheim, Germany; the TUH cohort). The lower level of quantitation was either 0.2 µg/L (from 2012 to 2018) and 1.8 µg/L (from 2019 to 2020) for the TAUH cohort, and 0.3 µg/L for the TUH cohort, the intra-assay CVs were 1.1% and 2.8%, and the inter-assay CVs were 2.6% and 3.3%, respectively. Serum CA19-9 was analysed with an immunoenzymatic assay from Roche Cobas (Pleasanton, California, USA), and serum CRP was analysed using an immunoturbidimetric method (Roche Diagnostics, Mannheim, Germany). The lower levels of quantitation for the serum CA19-9 and CRP were 3 kU/L and 0.6 mg/L, the intra-assay CVs were 1.2% and 1.8%, and the inter-assay CVs were 2.1% and 2.5%, respectively. All the measurements were performed by technicians that were blinded to the study’s endpoints.

### 4.4. Biomarker Cut-Offs

Serum CEA levels of >5 µg/L, CA19-9 levels of >26 kU/L, and CRP levels of >10 mg/L were considered to be elevated [5,53]. The median serum IL-6 concentration in healthy subjects is 1.3 pg/mL, with a 2.5% to 97.5% reference range from 0.33 pg/mL to 26 pg/mL [54]. Therefore, we considered serum IL-6 levels that were greater than the 95th percentile level to be elevated in healthy individuals (>4.5 pg/mL).

The median serum YKL-40 concentration is 40 ng/mL in healthy subjects (2.5% to 97.5% reference range, 14 ng/mL to 155 ng/mL) [53]. An elevated serum YKL-40 concentration was defined as a value above the 90th percentile of the age-adjusted healthy controls, where the age-adjusted percentile of normal was calculated as a function of age in years and the serum YKL-40 in ng/mL, using the formula: percentile = 100/(1 + (YKL-40 ^−3)*(1.062^age)*5000). We used the raw (unadjusted) values to evaluate the YKL-40 serum concentration changes during treatment.

### 4.5. Biomarker Changes with Time

The patients were grouped into three groups, according to the longitudinal kinetic patterns in the serum biomarker concentrations. The grouping was performed blinded to the survival outcome s.

We first compared the biomarker concentrations between the baseline (pre-adjuvant) samples and the mid-adjuvant samples (Figure S1). The biomarker concentrations that were ≥10% higher in the mid-adjuvant sample compared to the baseline were classified as increased concentrations, whereas the patients who had an increase of <10%, a stable concentration, or any decrease in their biomarker concentrations were classified as “no increase”. A 10% cut-off was chosen, because a ≥10% change was greater than the intra-assay and inter-assay coefficients of variation (CV) of the tumour markers that were evaluated, and the 10% cut-off did not depend on the biomarker reference range. When the biomarker concentration was, by any amount, smaller in the post-adjuvant sample than the increased biomarker concentration in the mid-adjuvant sample, we denoted the increase as “a transient increase”, otherwise the increase was classified as a “persistent increase”.

### 4.6. Statistical Analyses

The clinicopathological parameters and biomarker values were presented as frequencies or medians with a range for nonparametric distributions. The chi-squared test was used for comparisons between the categorical variables, and the Mann–Whitney test or the Kruskal–Wallis test for comparing the non-normally distributed continuous variables. The OS and DFS were estimated with the Kaplan–Meier method. The DFS was defined as the time period from the date of randomisation or surgery to the date of recurrence or death from any cause, with censoring for the patients that were alive without a recurrence on the last date of follow-up. The OS was defined as the time period from the date of randomisation or surgery to the date of death from any cause, with censoring for the patients that were alive on the last date of follow-up. The patients with an event (a recurrence or death) during the first 8 months of follow-up were removed from the long-term analysis, since the event occurred during the time frame of interest, i.e., during adjuvant-treatment-induced changes. The adjusted hazard ratios (HRs) and 95% confidence intervals (CI) were estimated with the Cox regression proportional hazard model. Adjustments were made for the tumour–node–metastasis (TNM) stage, age, sex, chemotherapy regimen, radiotherapy, immune-related comorbidity, and primary tumour location in the adjusted analyses for all the cohorts, and, additionally, for the tumour grade and Charlson comorbidity index in the TAUH and TUH cohorts, and for smoking in the TUH cohort. Immune-related comorbidities possibly affecting CRP, YKL-40, and IL-6 levels were rheumatoid arthritis, iritis, psoriasis, non-active inflammatory bowel disease, and a thyroiditis in the history. The median follow-up time was calculated by the reverse Kaplan–Meier method. The statistical significance level was set at 5%; all the tests were two-sided. The statistical analyses were performed with SPSS version 27.0 (IBM SPSS Statistics, version 27.0 for Mac; SPSS, Inc., Chicago, IL, USA).

## 5. Conclusions

In conclusion, we found that a transient increase in serum CEA is associated with favourable survival outcomes in patients that are treated with adjuvant chemotherapy for CRC. These low-cost markers may also have predictive value. The current findings suggest that the change patterns of CEA, CA19-9, and YKL-40 during adjuvant chemotherapy can predict CRC recurrence and patient survival. There are no metrics for the evaluation of the chemotherapy response in the adjuvant setting, but the monitoring of serum CEA during adjuvant therapy might be helpful when personalizing patient follow-up. The evaluation of the CEA change patterns and of other biomarkers, such as CA19-9 and YKL-40, in the follow-up of patients with CRC and other types of human cancer, warrants further study.

## Figures and Tables

**Table 1:** Patient and tumour characteristics.

Characteristics	LIPSYT Patients	TAUH Patients	TUH Patients
	n = 131	n = 346	n = 281
Age, median years (range)	60 (31–76)	67 (33–87)	67 (28–84)
Age group, years (%)			
<70	116 (89)	217 (63)	178 (64)
≥70	15 (12)	129 (37)	101 (36)
Sex			
Male	70 (53)	191 (55)	149 (53)
Female	61 (47)	155 (45)	130 (47)
Inflammatory disease ^a^			
No	117 (89)	329 (95)	266 (95)
Yes	14 (11)	17 (5)	13 (5)
Charlson comorbidity index			
0	-	205 (59)	224 (80)
1	-	107 (31)	38 (80)
2 or more	-	34 (10)	17 (6)
Smoking			
Never	-	-	155 (56)
Former/current	-	-	107 (38)
Unknown	-	-	17 (6)
Primary location			
Right colon	38 (29)	105 (30)	68 (24)
Left colon	41 (31)	97 (28)	70 (25)
Rectal	52 (40)	144 (42)	141 (51)
TNM stage ^b^			
IIA-B	38 (29)	84 (24)	90 (32)
IIIA-C	82 (63)	259 (75)	186 (67)
IV	11 (8)	3 (1)	3 (1)
Grade			
I-II	-	251 (72%)	212 (76)
III	-	95 (28)	67 (24)
Radiotherapy for rectal primary			
No	7 (13)	41 (28)	57 (40)
Preoperative 5 × 5 Gy	8 (15)	40 (28)	50 (35)
Chemoradiation ^c^	37 (71)	63 (44)	34 (24)
Chemotherapy regimen			
5-FU + LV bolusinj ^d^	66 (50)	-	-
5-FU + LV continuous inf	65 (50)	-	-
Capecitabine	-	155 (45)	120 (43)
Capecitabine + oxaliplatin ^e^	-	190 (55)	159 (57)

^a^ Inflammatory diseases adjusted for: autoimmune diseases as rheumatoid arthritis, iritis, psoriasis, ulcerative colitis, coeliac disease, and thyroiditis. ^b^ ypTNM stage in patients who had preopeative chemoradiation in the TUH data. ^c^ Preoperative chemoradiation in TAUH and TUH and mostly postoperative in LIPSYT. ^d^ 5FU + LV = 5-fluorouracil and leucovorin with randomization to bolus injection (Mayo regimen) or continuous infused (de Gramont regimen). ^e^ at least one cycle of oxaliplatin.

**Table 2:** Biomarker dynamics during adjuvant chemotherapy in the three cohorts with name marked in bold. Cut-off values: CEA > 5 µg/L, CA19-9 > 26 kU/L, YKL-40 > 90th percentile at baseline, CRP > 10 mg/L, and IL-6 > 4.5 pg/mL.

Biomarker	All	Transient Increase	No Increase	Persistent Increase
**LIPSYT study**				
CEA				
Patients, n (%)	91	42 (46)	32 (35)	17 (19)
0 months, Median (range) (µg/L)	1.3 (0–4.5)	1.6 (<0.5–3.8)	1.0 (0–4.5)	1.4 (<0.5–2.8)
4 months, Median (range) (µg/L)	2 (<0.5–6.6)	2.4 (1.0–6.6)	0.9 (<0.5–4.3)	2.2 (0.6–4.5)
8 months, Median (range) (µg/L)	1.6 (<0.5–7.3)	1.8 (<0.5–4.1)	1.0 (<0.5–5.8)	2.6 (0.9–7.3)
CA19-9				
Patients, n (%)	50	6 (12)	38 (76)	6 (12)
0 months, Median (range) (kU/L)	<5 (<5–26)	7 (<5–16)	<5 (<5–26)	6 (<5–22)
4 months, Median (range) (kU/L)	<5 (4–36)	9 (6–27)	<5 (4–20)	8 (5–34)
8 months, Median (range) (kU/L)	6 (4–333)	8 (4–24)	<5 (4–25)	13 (6–333)
CRP				
Patients, n (%)	119	21 (18)	95 (80)	3 (3)
0 months, Median (range)(mg/L)	4 (4–10)	4 (4–10)	4 (4–10)	4 (4–6)
4 months, Median (range)(mg/L)	4 (4–70)	19 (7–70)	4 (4–8)	7 (7–10)
8 months, Median (range)(mg/L)	4 (1–15)	4 (4–13)	4 (1–15)	10 (7–15)
YKL-40				
Patients, n (%)	92	52 (57)	33 (36)	7 (8)
0 months, Median (range) (µg/L)	49 (20–140)	47 (20–130)	46 (20–140)	55 (38–115)
4 months, Median (range) (µg/L)	70 (20–267)	100 (28–267)	45 (20–110)	87 (61–125)
8 months, Median (range) (µg/L)	56 (20–217)	56 (20–217)	44 (20–189)	122 (68–176)
IL-6				
Patients, n (%)	97	42 (43)	44 (45)	11 (11)
0 months, Median (range) (µg/L)	2.0 (0.4–4.5)	1.8 (0.4–4.5)	2.3 (0.9–4.1)	2.0 (0.8–3.9)
4 months, Median (range) (µg/L)	2.5 (0.6–24.9)	3.9 (0.8–24.9)	1.8 (0.6–3.4)	2.6 (1.1–6.6)
8 months, Median (range) (µg/L)	1.7 (0.2–14.0)	1.7 (0.7–5.7)	1.7 (0.2–14.0)	3.1 (1.6–11.3)
**TAUH cohort**				
CEA				
Patients, n (%)	346	176 (51)	72 (21)	98 (28)
0 months, Median (range) (µg/L)	1.8 (0.3–5)	1.8 (0.3–4.9)	1.8 (0.7–4.9)	1.8 (0.5–5)
4 months, Median (range) (µg/L)	2.8 (0.5–245)	3.3 (0.8–28.2)	1.8 (0.5–4.7)	3.0 (0.6–245)
8 months, Median (range) (µg/L)	2.3 (0.5–655)	2.2 (0.6–7.9)	1.8 (0.5–45.3)	3.2 (0.7–655)
CA19-9				
Patients, n (%)	104	48 (46)	30 (29)	26 (25)
0 months, Median (range) (kU/L)	7 (<5–24)	8 (<5–20)	6 (<5–23)	8 (<5–24)
4 months, Median (range) (kU/L)	11 (<5–112)	12 (6–63)	5 (<5–24)	13 (6–122)
8 months, Median (range) (kU/L)	8 (<5–2748)	8 (<5–49)	5 (<5–57)	14 (6–2748)
CRP				
Patients, n (%)	283	25 (9)	237 (84)	21 (7)
0 months, Median (range)(mg/L)	1 (1–10)	1 (1–5)	1 (1–10)	1 (1–4)
4 months, Median (range)(mg/L)	1 (1–12)	3 (1–10)	1 (1–7)	3 (1–12)
8 months, Median (range)(mg/L)	1 (1–255)	1 (1–6)	1 (1–255 ^a^)	6 (1–78)
**TUH cohort**				
CEA				
Patients, n (%)	279	197 (71)	32 (11)	50 (18)
0 months, Median (range)(µg/L)	1.6 (0.2–5)	1.5 (0.2–4.9)	2.8 (0.9–5)	1.5 (0.3–4.5)
4 months, Median (range)(µg/L)	2.9 (0.6–23)	3.1 (0.9–23)	2.3 (0.8–5)	2.8 (0.6–19)
8 months, Median (range)(µg/L)	2.2 (0.2–38)	2.0 (0.2–12)	2.0 (0.4–8.4)	3.0 (0.6–38)

^a^ Infection value. Cut-off values: CEA >5 µg, CA19-9 >26 kU/L, YKL-40 >90th percentile, CRP >10 mg/L, and IL-6 > 4.5 pg/mL. Exclusions and missing data presented in Figure S1A,B.

**Table 3:** 10-year DFS and OS rates in the LIPSYT study according to biomarker dynamics during adjuvant therapy.

Biomarker	All	Transient Increase	No Increase	Persistent Increase
**CEA**				
Patients, n (%)	91	42 (46)	32 (35)	17 (19)
Relapses, n (%)	20 (22)	7 (17)	5 (16)	8 (47)
Non-CRC deaths, n (%)	9 (10)	4 (10)	5 (16)	0 (0)
Deaths (any cause)	27 (30)	10 (24)	9 (28)	8 (47)
10-year DFS rate	70%	79%	69%	53%
10-year OS rate	73%	81%	72%	53%
**CA19-9**				
Patients, n (%)	50	6 (12)	38 (76)	6 (12)
Relapses, n (%)	23 (46)	0 (0)	19 (50)	4 (67)
Non-CRC deaths, n (%)	7 (14)	1 (17)	6 (16)	0 (0)
Deaths (any cause)	27 (54)	1 (17)	22 (58)	4 (67)
10-year DFS rate	42%	83%	37%	33%
10-year OS rate	46%	83%	42%	33%
**CRP**				
Patients, n (%)	119	21 (18)	95 (80)	3 (3)
Relapses, n (%)	44 (37)	6 (29)	36 (38)	2 (67)
Non-CRC deaths, n (%)	11 (9)	2 (10)	8 (8)	1 (33)
Deaths (any cause)	50 (42)	8 (38)	39 (41)	3 (100)
10-year DFS rate	56%	62%	57%	0%
10-year OS rate	61%	62%	63%	0%
**YKL-40**				
Patients, n (%)	92	52 (57)	33 (36)	7 (8)
Relapses, n (%)	35 (28)	14 (27)	15 (45)	6 (86)
Non-CRC deaths, n (%)	8 (9)	7 (13)	1 (3)	0 (0)
Deaths (any cause)	39 (42)	20 (38)	14 (42)	5 (71)
10-year DFS rate	54%	62%	52%	14%
10-year OS rate	59%	62%	57%	43%
**IL-6**				
Patients, n (%)	97	42 (43)	44 (45)	11 (11)
Relapses, n (%)	38 (39)	15 (36)	17 (39)	6 (55)
Non-CRC deaths, n (%)	9 (9)	4 (10)	3 (7)	2 (18)
Deaths (any cause)	44 (45)	19 (45)	18 (41)	7 (64)
10-year DFS rate	54%	57%	57%	27%
10-year OS rate	58%	57%	64%	36%

**Table 4:** Disease-free survival (DFS) and overall survival (OS) according to biomarker dynamics within reference during adjuvant therapy. Adjusted hazard ratio (HR) and 95% confidence interval (CI), with persistent increase in biomarker level used as reference groups.

		Transient Increase		No Increase		Persistent Increase
		HR ^a^	95% CI	HR ^a^	95% CI	
**LIPSYT study**						
CEA ^b^	DFS	0.21	0.07–0.66	0.29	0.09–0.92	Reference
	OS	0.24	0.08–0.76	0.33	0.10–1.05	
CA19-9 ^c^	DFS	0.13	0.01–1.52	0.68	0.20–2.33	Reference
	OS	0.11	0.01–1.22	0.67	0.20–2.27	
CRP ^d^	DFS	0.27	0.07–1.08	0.33	0.09–1.16	Reference
	OS	0.25	0.06–1.01	0.30	0.08–1.06	
YKL-40 ^e^	DFS	0.21	0.06–0.73	0.34	0.10–1.12	Reference
	OS	0.25	0.07–0.92	0.40	0.11–1.43	
IL-6 ^f^	DFS	0.40	0.16–1.02	0.40	0.16–1.02	Reference
	OS	0.52	0.20–1.36	0.46	0.17–1.19	
**TAUH cohort**						
CEA ^g^	DFS	0.23	0.14–0.38	0.88	0.51–1.51	Reference
	OS	0.32	0.17–0.61	0.64	0.30–1.34	
CA19-9 ^h^	DFS	0.22	0.07–0.67	0.39	0.21–1.28	Reference
	OS	0.10	0.02–0.65	0.19	0.03–1.18	
CRP ^i^	DFS	0.35	0.09–1.39	0.90	0.04–1.99	Reference
	OS	0.70	0.12–4.03	1.13	0.39–3.24	
**TUH cohort**						
CEA ^j^	DFS	0.39	0.22–0.66	0.18	0.09–0.92	Reference
	OS	0.14	0.06–0.33	0.05	0.01–0.43	

^a^ Adjusted with TNM stage, age, sex, chemotherapy treatment, radiation therapy, inflammatory disease, and primary tumour location in all cohorts, grade and Charlson comorbidity index in TAUH and TUH cohorts, and smoking only in TUH cohort. ^b^ CEA n = 91, ^c^ CA19-9 n = 50, ^d^ CRP n = 119, ^e^ YKL-40 n = 92, ^f^ IL-6 n = 97, ^g^ CEA n = 346, ^h^ CA19-9 = 104, ^i^ CRP = 283, and ^j^ CEA n = 279.

## Data Availability

The data collected for this study and cohorts can be made available to others in de-identified form after all primary and secondary endpoints have been published, in the presence of a data transfer agreement, and if the purpose of use complies with Finnish legislation. Requests for data sharing can be made to the corresponding author, including a proposal that must be approved by the steering committee.

## Supplementary Materials

The following supporting information can be downloaded at: https://www.mdpi.com/article/10.3390/ijms24076753/s1.
