# Supplementary material for: Transient Changes in Serum CEA, CA19-9, CRP, YKL-40, and IL-6 during Adjuvant Chemotherapy and Survival of Patients with Colorectal Cancer
Source: Int J Mol Sci. 2023 Apr 4;24(7):6753. doi: 10.3390/ijms24076753 (PMC10094778; doi:10.3390/ijms24076753)
Supplement: Supplementary file 1 [file ijms-24-06753-s001.zip › ijms-2255035-supplementary.pdf]

SUPPLEMENTARY CONTENTS

Figure S1A. Flow diagram showing the selection of patients, categorization of pattern of biomarker change, and inclusion in analyses for CEA in LIPSYT / TAUH / TUH cohorts................................2

Figure S1B. Flow diagram showing the selection of patients, categorization of pattern of biomarker change, and inclusion in analyses for CA19-9, CRP, YKL-40, IL-6 in the LIPSYT cohort...............3

Figure S2. CEA dynamics within reference range during adjuvant therapy per patient in the LIPSYT data 4

Figure S3. Disease-free survival (DFS) (LIPSYT study panel A. TAUH panel C) and overall survival (OS) (LIPSYT study panel B and TAUH cohort panel D) according to CA19-9 kinetics during adjuvant therapy 5

Figure S4. Disease-free survival (DFS) (LIPSYT study panel A. TAUH panel C) and overall survival (OS) (LIPSYT study panel B and TAUH cohort panel D) according to CRP kinetics during adjuvant therapy..6

Figure S5. Disease-free survival (DFS) (panel A) and overall survival (OS) (panel B) according to YKL-40 kinetics during adjuvant therapy 7

Figure S6. Disease-free survival (DFS) (panel A) and overall survival (OS) (panel B) according to IL-6 kinetics during adjuvant therapy..8

Table S1. Patient demographics according to CEA dynamics group. Statistically significant results are presented in red 9

Table S2. Associations of "Transient increase", "No increase" and "Persistent increase" of CEA with haematological and biochemical parameters, and treatment-related adverse events................10

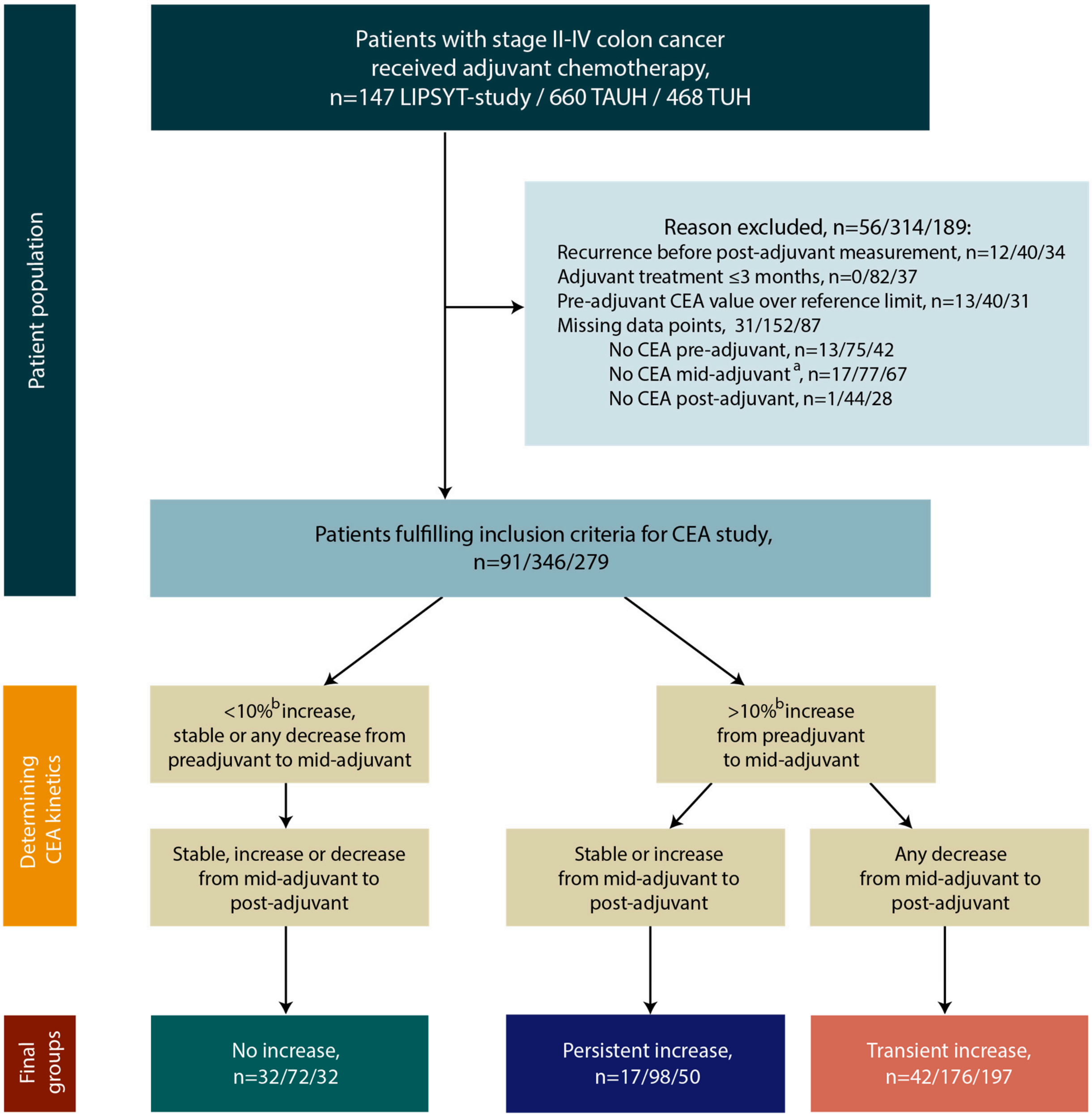

[a] CEA was not routinely measured in the early inclusion period in any of the cohorts.
[b] The 10% cut-off was chosen, because a >10% change was greater than the intra-assay and inter-assay coefficient of variation (CV) of the biomarkers evaluated, and the 10% cut-off does not depend on the biomarker reference range

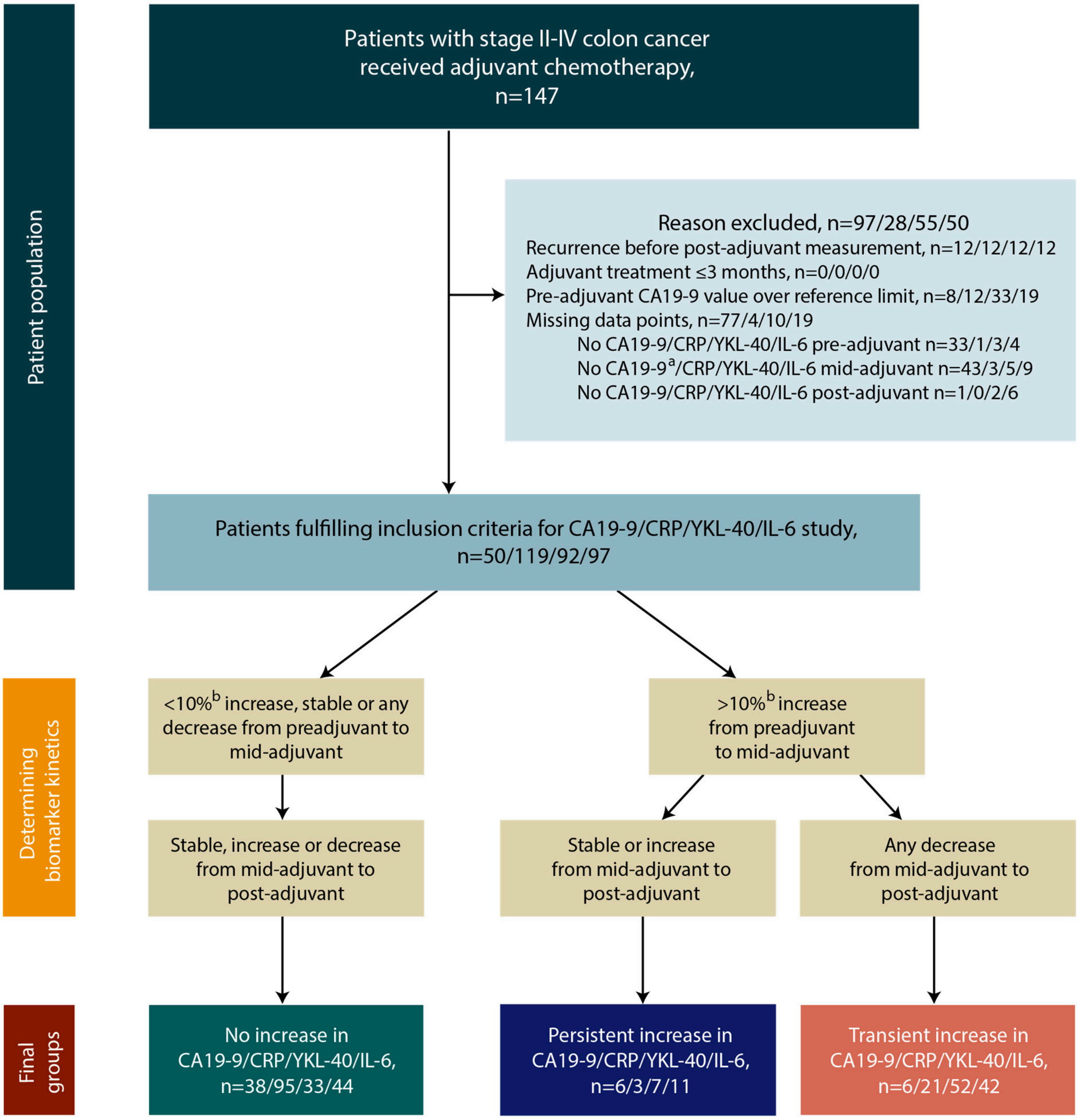

[a] CA19-9 was not routinely measured in the early inclusion period in the LIPSYT-study
[b] The 10% cut-off was chosen, because a >10% change was greater than the intra-assay and inter-assay coefficient of variation (CV) of the biomarkers evaluated, and the 10% cut-off does not depend on the biomarker reference range

Figure S1. A. Flow diagram showing the selection of patients, categorization of pattern of biomarker change, and inclusion in analyses for CEA in LIPSYT / TAUH / TUH cohorts. B. Flow diagram showing the selection of patients, categorization of pattern of biomarker change, and inclusion in analyses for CA19-9, CRP, YKL-40, IL-6 in the LIPSYT cohort.

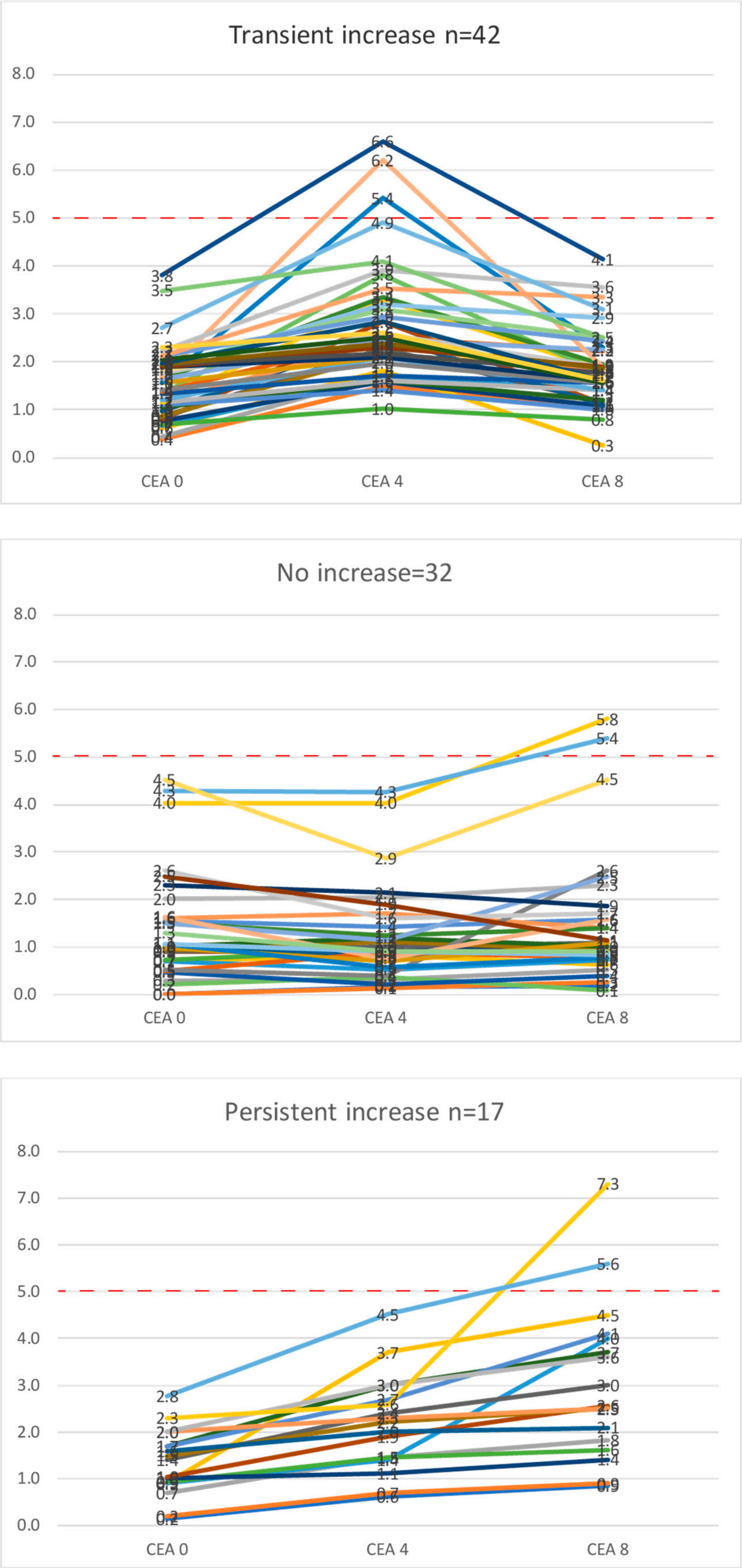

Figure S2. CEA dynamics within reference range during adjuvant therapy per patient in the LIPSYT data.

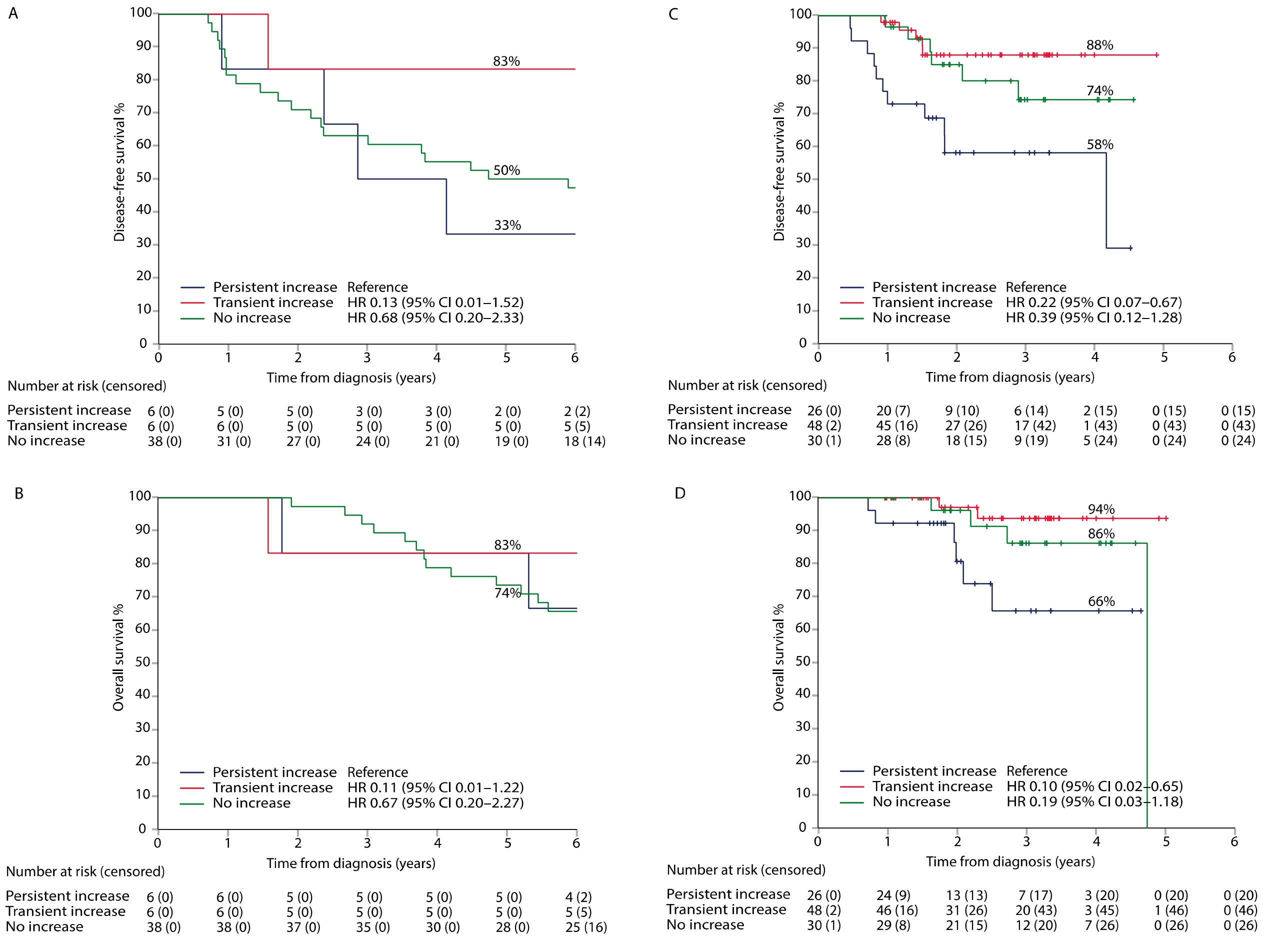

Figure S3. Disease-free survival (DFS) (LIPSYT study panel A. TAUH panel C) and overall survival (OS) (LIPSYT study panel B and TAUH cohort panel D) according to CA19-9 kinetics during adjuvant therapy.

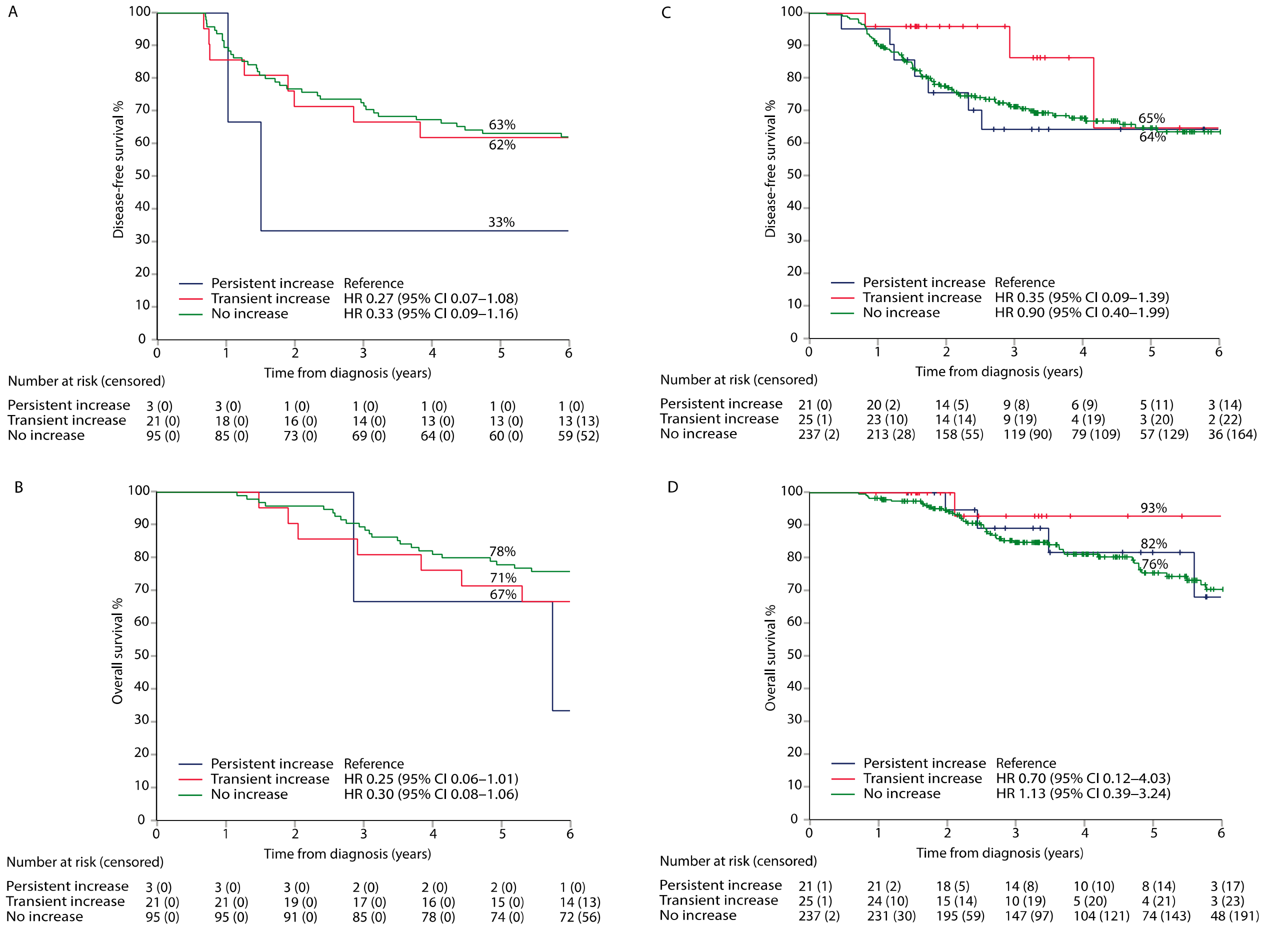

Figure S4. Disease-free survival (DFS) (LIPSYT study panel A. TAUH panel C) and overall survival (OS) (LIPSYT study panel B and TAUH cohort panel D) according to CRP kinetics during adjuvant therapy.

A

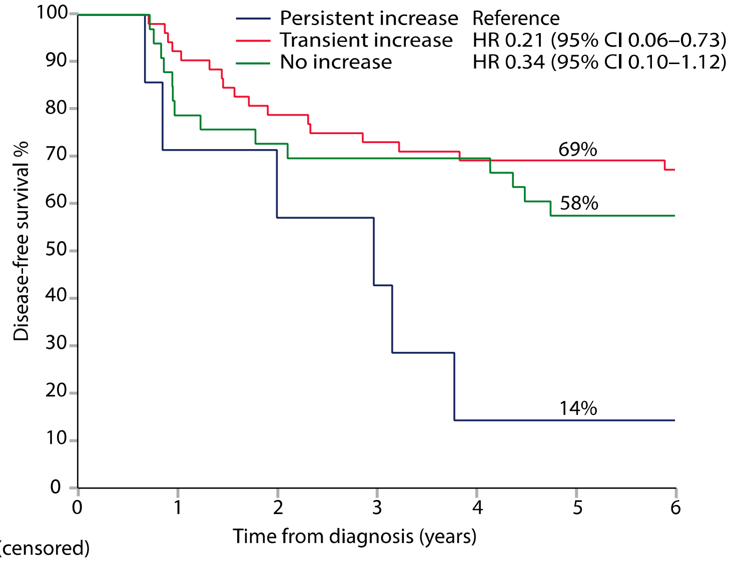

Number at risk (censored)

Persistent increase	7 (0)	5 (0)	5 (0)	3 (0)	1 (0)	1 (0)	1 (1)
Transient increase	52 (0)	48 (0)	41 (0)	38 (0)	36 (0)	36 (0)	35 (32)
No increase	33 (0)	26 (0)	24 (0)	23 (0)	23 (0)	19 (0)	19 (17)

B

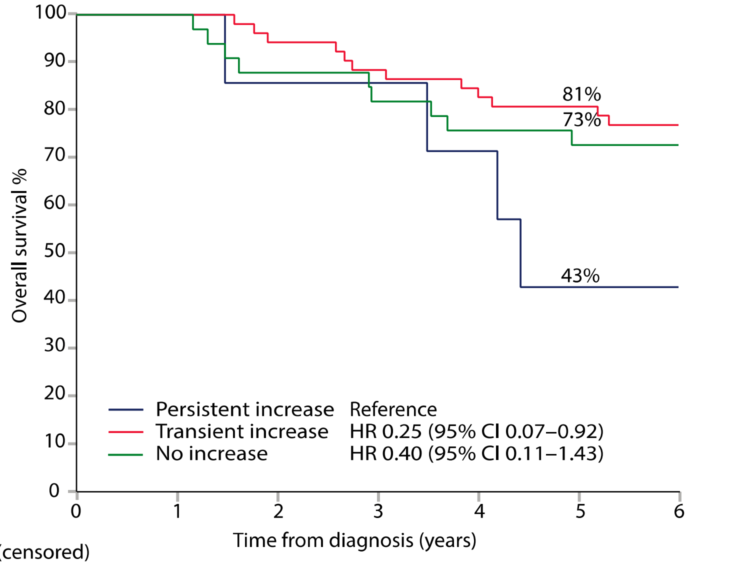

Number at risk (censored)

Persistent increase	7 (0)	7 (0)	6 (0)	6 (0)	5 (0)	3 (0)	3 (2)
Transient increase	52 (0)	52 (0)	49 (0)	46 (0)	44 (0)	42 (0)	40 (32)
No increase	33 (0)	33 (0)	29 (0)	27 (0)	25 (0)	24 (0)	24 (19)

Figure S5. Disease-free survival (DFS) (panel A) and overall survival (OS) (panel B) according to YKL-40 kinetics during adjuvant therapy.

A

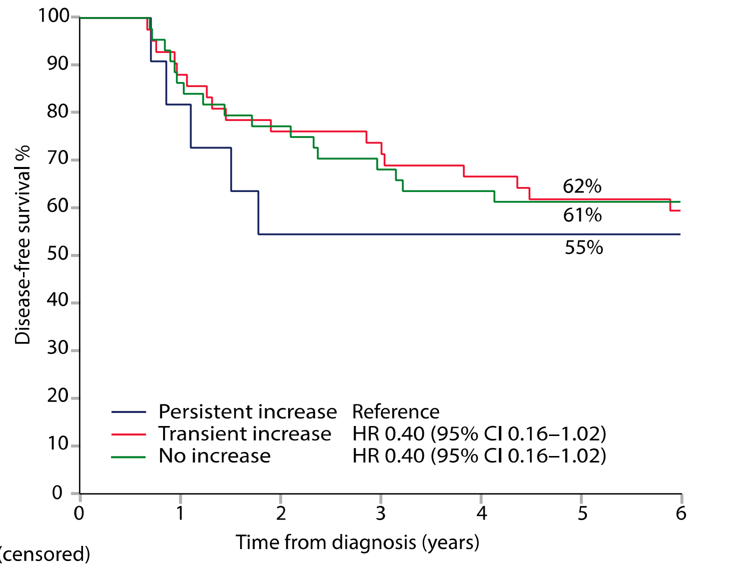

Number at risk (censored)

	0	1	2	3	4	5	6
Persistent increase	11 (0)	9 (0)	6 (0)	6 (0)	6 (0)	6 (0)	6 (3)
Transient increase	42 (0)	37 (0)	32 (0)	31 (0)	28 (0)	26 (0)	25 (23)
No increase	44 (0)	38 (0)	34 (0)	30 (0)	28 (0)	27 (0)	27 (24)

B

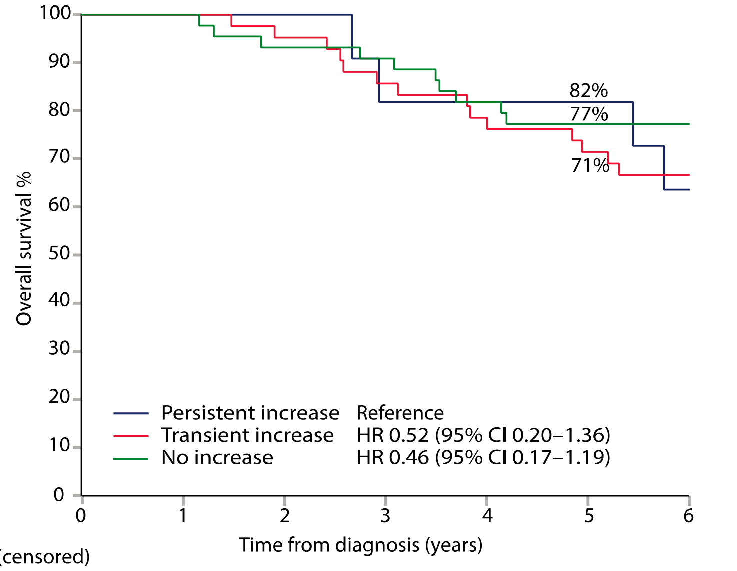

Number at risk (censored)

	0	1	2	3	4	5	6
Persistent increase	11 (0)	11 (0)	11 (0)	9 (0)	9 (0)	9 (0)	7 (4)
Transient increase	42 (0)	42 (0)	40 (0)	36 (0)	33 (0)	30 (0)	28 (23)
No increase	44 (0)	44 (0)	41 (0)	40 (0)	36 (0)	34 (0)	34 (26)

Figure S6. Disease-free survival (DFS) (panel A) and overall survival (OS) (panel B) according to IL-6 kinetics during adjuvant therapy.

Table S1. Patient demographics according to CEA dynamics group. Statistically significant results are presented in red.

		LIPSYT study						TAUH cohort						TUH cohort					
		Transient increase		No increase		Persistent increase		Transient increase		No increase		Persistent increase		Transient increase		No increase		Persistent increase	
		n=42	46 %	n=32	35 %	n=17	19 %	n=176	51 %	n=72	21 %	n=98	28 %	n=197	71 %	n=32	11 %	n=50	18 %
Age	Median yrs (range)	61 (37–74)		58 (50–73)		61 (31–70)		67 (24–85)		68 (37–87)		68 (39–85)		67 (33–84)		68 (50–83)		65 (28–81)	
	<70	35	83 %	30	94 %	17	100 %	117	67 %	42	58 %	58	59 %	123	62 %	21	66 %	34	68 %
	≥70	7	17 %	2	6 %	0	0 %	59	34 %	30	42 %	40	41 %	74	38 %	11	34 %	16	32 %
Sex	Male	21	50 %	17	53 %	9	53 %	90	51 %	44	61 %	57	58 %	105	53 %	11	34 %	33	66 %
	Female	21	50 %	15	47 %	8	47 %	86	49 %	28	39 %	41	42 %	93	47 %	21	66 %	17	34 %
Inflammatory disease*	No	40	95 %	28	88 %	15	88 %	167	95 %	66	92 %	96	98 %	189	96 %	29	91 %	48	96 %
	Yes	2	5 %	4	13 %	2	12 %	9	5 %	6	8 %	2	2 %	8	4 %	3	9 %	2	4 %
Chemotherapy regimen	5FU+LV bolusinj	23	55 %	20	63 %	4	24 %							-	-	-	-	-	-
	5FU+LV continuous inf	19	45 %	12	38 %	13	77 %							-	-	-	-	-	-
	capecitabine	-	-	-	-	-	-	58	33 %	44	62 %	53	54 %	76	39 %	16	50 %	28	56 %
	capecitabine+oxaliplatin	-	-	-	-	-	-	118	67 %	27	38 %	45	46 %	121	61 %	16	50 %	22	44 %
Primary location	Right colon	11	26 %	5	16 %	8	47 %	64	36 %	18	25 %	26	27 %	97	49 %	14	44 %	8	16 %
	Left colon	18	43 %	8	25 %	3	18 %	49	27 %	17	23 %	31	32 %	47	24 %	11	34 %	12	24 %
	Rectal	13	31 %	19	59 %	6	35 %	66	37 %	38	52 %	41	42 %	53	27 %	7	22 %	30	60 %
Radiotherapy for rectal primary	No	3	23 %	2	11 %	0	0 %	135	77 %	42	58 %	66	67 %	141	72 %	26	81 %	28	56 %
	pre op	2	15 %	2	11 %	2	33 %	14	8 %	12	17 %	14	14 %	33	17 %	5	16 %	12	24 %
	post op/chemoradiation	8	62 %	15	79 %	4	67 %	27	15 %	18	25 %	18	18 %	23	12 %	1	3 %	10	20 %
(y)pTNM stage	IIA-B	16	38 %	14	44 %	3	18 %	35	20 %	23	32 %	26	27 %	63	32 %	9	28 %	18	36 %
	IIIA-C	24	57 %	16	50 %	13	77 %	140	80 %	48	67 %	71	72 %	134	68 %	21	66 %	31	62 %
	IV	2	5 %	2	6 %	1	6 %	1	1 %	1	1 %	1	1 %	0	0 %	2	6 %	1	2 %
Gradus	I-II	-	-	-	-	-	-	130	74 %	50	69 %	71	72 %	152	77 %	25	78 %	35	70 %
	III	-	-	-	-	-	-	46	26 %	22	31 %	27	28 %	45	23 %	7	22 %	15	30 %
Smoking	never	-	-	-	-	-	-	-	-	-	-	-	-	109	55 %	22	69 %	24	48 %
	former/current	-	-	-	-	-	-	-	-	-	-	-	-	78	39 %	8	25 %	21	42 %
	Unknown	-	-	-	-	-	-	-	-	-	-	-	-	10	5 %	2	6 %	5	10 %
	0	-	-	-	-	-	-	111	63 %	41	57 %	53	54 %	163	83 %	24	75 %	37	74 %
Charlson comorbidity index	1	-	-	-	-	-	-	53	30 %	21	29 %	33	34 %	21	11 %	6	19 %	11	22 %
	2 or more	-	-	-	-	-	-	12	7 %	10	14 %	12	12 %	13	7 %	2	6 %	2	4 %

Table S2. Associations of "Transient increase", "No increase" and "Persistent increase" of CEA with haematological and biochemical parameters, and treatment-related adverse events.

	Transient increase<br>n = 42	No increase<br>n = 32	Persistent increase<br>n = 17	
**Laboratory test - median (range)**				p-value[a]
Alanine amino transferase	0 (-336 - +22)	1 (-47 - +86)	2 (-26 - +11)	0.923
Alkaline phosphatase	6 (-260 - +105)	-4 (-43 - +39)	4 (-230 - +480)	0.642
Creatinine	4 (-10 - +25)	1 (-33 - +18)	7 (-3 - +26)	0.029/0.514[b]
Sodium	0 (-6 - +9)	-0.5 (-6 - +4)	0 (-3 - +6)	0.374
Potassium	0 (-1.5 - +0.6)	0 (-0.6 - +0.5)	0 (-0.3 - +0.5)	0.524
INR	0 (-0.2 - +0.91)	0 (-0.1 - +2)	0 (-0.1 - +0.7)	0.679
Tromboplastin time	-9 (-62 - +101)	-15 (-62 - +31)	-5 (-58 - +44)	0.639
Prealbumin	20 (-53 - +92)	14 (-90 - +105)	10 (-39 - +85)	0.745
Albumin	-0.3 (-12 - +7.6)	-1.6 (-12.2 - +5.6)	0.5 (-6.5 - +6.6)	0.157
Haemoglobin	0 (-34 - +16)	-2.5 (-17 - +14)	1 (-15 - +43)	0.283
Leukocytes	-0.6 (-5.1 - +2.5)	-1.2 (-13.8 - +1.8)	-1.4 (-2.9 - +0.29)	0.064
Trombocytes	2.5 (-154 - +54)	0 (-19 - +66)	1 (-66 - +12)	0.133
**Adverse events - grade 0-1/2-4 (%)**				p value
Worst any toxicity	76% / 24%	75% / 25%	77% / 24%	0.991
Worst haematological toxicity	52% / 48%	28% / 72%	59% / 41%	0.052
Diarrhoea	43% / 57%	31% / 69%	47% / 53%	0.469
Stomatitis	36% / 64%	41% / 59%	65% / 35%	0.119
Mucositis	91% / 10%	84% / 16%	100% / 0%	0.217
Flatulence	95% /5%	91% / 9%	100% / 0%	0.375
Heartburn	91% / 10%	81% / 19%	94% / 6%	0.331

[a] Kruskal-Wallis for delta (mid-adjuvant minus before adjuvant) of laboratory value in CEA kinetics groups.
[b] Chi-Square for "transient increase", "no Increase", vs. "persistent increase" kinetics of CEA and creatinine.
